# Comparative Analysis of Human B Cell Epitopes Based on BCG Genomes

**DOI:** 10.1155/2016/3620141

**Published:** 2016-06-12

**Authors:** Machao Li, Haican Liu, Xiuqin Zhao, Kanglin Wan

**Affiliations:** State Key Laboratory of Infectious Diseases Prevention and Control, Collaborative Innovation Center for Diagnosis and Treatment of Infectious Diseases, National Institute for Communicable Disease Control and Prevention, Chinese Center for Disease Control and Prevention, Beijing 102206, China

## Abstract

*Background*. Tuberculosis is a huge global health problem. BCG is the only vaccine used for about 100 years against TB, but the reasons for protection variability in populations remain unclear. To improve BCG efficacy and develop a strategy for new vaccines, the underlying genetic differences among BCG subtypes should be understood urgently.* Methods and Findings*. Human B cell epitope data were collected from the Immune Epitope Database. Epitope sequences were mapped with those of 15 genomes, including 13 BCGs,* M. bovis* AF2122/97, and* M. tuberculosis* H37Rv, to identify epitopes distribution. Among 398 experimentally verified B cell epitopes, 321 (80.7%) were conserved, while the remaining 77 (19.3%) were lost to varying degrees in BCGs. The variable protective efficacy of BCGs may result from the degree of B cell epitopes deficiency.* Conclusions*. Here we firstly analyzed the genetic characteristics of BCGs based on B cell epitopes and found that B cell epitopes distribution may contribute to vaccine efficacy. Restoration of important antigens or effective B cell epitopes in BCG could be a useful strategy for vaccine development.

## 1. Introduction

Tuberculosis (TB), mainly caused by* Mycobacterium tuberculosis* (*M. tuberculosis*), is an ancient scourge preceding the recorded human history [1]. Data released by World Health Organization (WHO) revealed that at least 9.6 million new cases would be recorded every year, with 1.5 million TB related deaths expected in 2014. It is estimated that the threat of TB would be aggravated by the impact of multidrug resistant TB (MDR-TB), extensively drug-resistant TB (XDR-TB), and TB/HIV coinfection [2, 3]. China is one of the 22 countries with high TB burden. In 2010, mortality due to all tuberculosis, pulmonary tuberculosis, and extrapulmonary tuberculosis was 4.69, 4.38, and 0.31 per 100 000 population, respectively [4].

Bacille Calmette-Guérin (BCG), an attenuated derivative of* Mycobacterium bovis *(*M. bovis*), which underwent 230 passages originally, has been the only permitted vaccine against TB for nearly a century worldwide. Despite debates focusing on its protective effects against TB [5, 6], BCG has been included in the WHO Expanded Program on Immunization, with 100 million administration cases annually.

It is widely accepted that protective immunity of humans against TB is mainly attributable to cell-mediated immunity, especially T lymphocytes. In addition, humoral immunity plays small role in the protective immunity to TB [7–9]. However, other scientists argue that B cells could work as antigen presenting cells (APC), secrete antibodies which play a regulatory role in T cell-mediated immune response, and produce cytokines [10–12]. In other words, B cells would yield more important effects than previously thought.

Even though BCG is the most widely used vaccine in human history, its mechanisms remain poorly understood. Additionally, there have been many daughter strains of BCG with varied protective efficacy [13, 14]. Here we performed comparative genomic analysis among BCGs to explain the protective differences and even determine more candidates for TB vaccine development from B cell epitopes.

## 2. Methods

### 2.1. Genome Sequences of BCG Strains

The 15 genome sequences assessed in this study belonged to 13 BCG strains (BCG-Moreau, BCG-Phipps, BCG-Sweden, BCG-China, BCG-Danish, BCG-Prague, BCG-Russia, BCG-Tice, BCG-Glaxo, BCG-Mexico, BCG-Pasteur, BCG-Tokyo 172, and BCG-Frappier),* M. tuberculosis* H37Rv, and* M. bovis* AF2122/97 [15–21]. A total of 6 BCG genomes were sequenced in our lab and results had been published, including BCG-Moreau, BCG-Phipps, BCG-Sweden, BCG-Prague, BCG-Glaxo, and BCG-Frappier [15]. The remaining genome sequences were acquired from National Center for Biotechnology Information (NCBI). All detailed information of the genome sequences is listed in Table S1 (see Supplementary Material available online at http://dx.doi.org/10.1155/2016/3620141).

### 2.2. Human B Cell Epitope Sequences

An online search in the Immune Epitope Database (IEDB) was performed, and peptidic epitopes were selected as targets in this study.

### 2.3. Confirmation of Nucleic Acid Sequence Coding Epitope

The sequences of peptidic epitopes were compared to the H37Rv proteome, using BLAST to confirm which genes encoded the amino acid sequences of various B cell epitopes [22]. If an epitope was located in more than one gene after mapping, the gene encoding that epitope was selected based on article which submitted this epitope. Upon gene identification, the nucleic acid sequence encoding the B cell epitope was confirmed by comparison with the amino acid sequence.

### 2.4. B Cell Epitope Distribution among BCG Strains and* M. bovis* AF2122/97

The gene sequences encoding B cell epitopes were compared individually with the whole genomes of BCG strains and* M. bovis* AF2122/97 to determine B cell epitope distribution in different strains. All deletion regions of DNA encoding B cell epitopes were validated by PCR and DNA sequencing.

## 3. Results

### 3.1. Peptidic B Cell Epitopes of* Mycobacterium tuberculosis* in the IEDB

A total of 399 experimentally verified peptidic human B cell epitopes were obtained in the IEDB. Since 2 epitopes (epitope IDs 94762 and 94763) shared the same nucleic acid sequence with only a few modified amino acids, epitope 94763 was not included in this study. Finally, 398 B cell epitopes involved were analyzed.

All peptidic epitopes were included in 81 genes according to* M. tuberculosis* H37Rv and* M. bovis* AF2122/97 genomes, respectively; epitopes and genes are shown in Table S2. Although the gene homologous to* Rv3347c* was split into 2 genes in* M. bovis* AF2122/97 genome, there were finally 81 genes encoding B cell epitopes in* M. bovis* AF2122/97 because of the absence of a gene homologous to* Rv2351c.*

### 3.2. Functional Classification of Genes Encoding B Cell Epitopes

**Figure 1:**
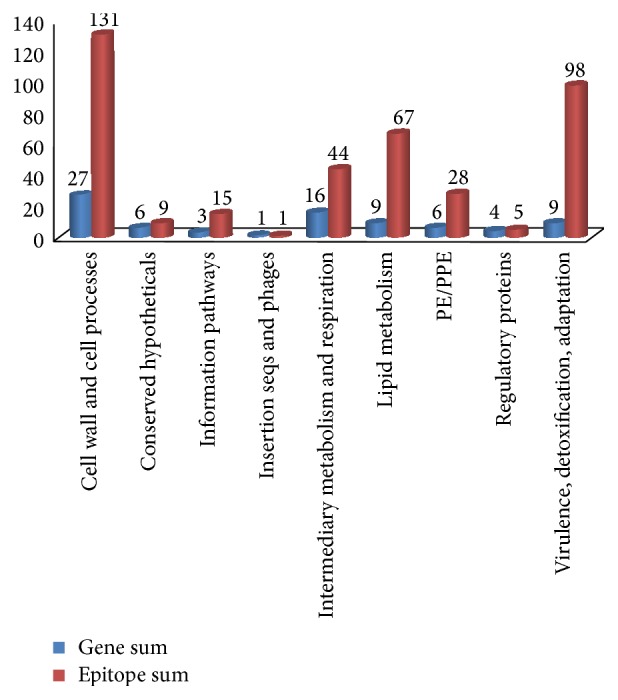
Distribution of epitopes in various functional categories.

According to an analysis by Cole et al. in 1998, all* M. tuberculosis* genes are classified into various categories based on function [23]. And functional classification could be searched online (*Tuberculist* http://tuberculist.epfl.ch/). In this study, the 81 genes belonged to 9 categories (Figure 1). The detailed information regarding the functional classification is summarized in Table S3. Genes for “cell and cell processes” and “virulence, detoxification, and adaptation” covered more than half of epitopes, and some important genes belonged to these 2 categories, such as* Rv3874* (encoding CFP-10) [24],* Rv3875* (encoding ESAT-6) [25],* Rv1980c* (encoding MPT-64) [24],* Rv0934 *(encoding PstS1) [26],* Rv0350* (encoding HSP70) [24], and* Rv0440* (encoding HSP65) [27].

### 3.3. Distribution of B Cell Epitopes in BCGs

**Figure 2:**
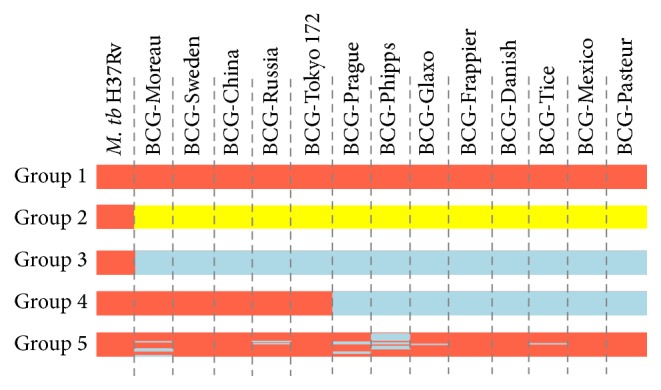
The composition of 398 human B cell epitopes distributed in BCGs. Five groups of B-cell epitopes in the 13 BCG strains. Epitopes shown in red were present, those shown in sky blue were absent, and epitopes labeled yellow were showing same SNPs in BCGs exclusively.

DNA fragments encoding B cell epitopes, based on H37Rv gene sequence, were extracted from the 13 BCG genomes to assess the changes of B cell epitopes. A total of 398 B cell epitopes were analyzed. No SNP was identified in 321 epitopes of all BCGs (classified as Group 1); the remaining 77 epitopes varied in DNA levels among BCGs to different degrees. Of the 77 nucleic acid-altered epitopes, 15 presented the same nucleotide change in all BCGs (classified as Group 2). A total of 26 epitopes were lost in all BCGs because of gene deletion (classified as Group 3). There were 13 epitopes lost in 8 BCG strains, including BCG-Phipps, BCG-Danish, BCG-Prague, BCG-Tice, BCG-Glaxo, BCG-Mexico, BCG-Pasteur, and BCG-Frappier (classified as Group 4). The remaining 23 epitopes showed different SNPs or nucleotide deletions in different BCGs (classified as Group 5) (Figure 2). B cell epitope grouping in BCGs is listed in Table S4.

Group 1 epitopes were present in all BCGs, with no SNP in these strains. In comparison with* M. bovis* AF2122/97 genome, only 2 of the 321 epitopes (epitope IDs 103022 and 103425) changed. The indicated changes likely derived from the original BCG, attenuated between 1908 and 1921 [14].

Group 2 included 15 epitopes showing the same SNPs in BCGs compared with* M. tuberculosis* H37Rv. Interestingly, 9 epitopes (epitope IDs 9924, 103007, 103038, 103061, 103266, 103352, 103579, 120511, and 120892) kept the same sequence in* M. bovis* AF2122/97 but differed in* M. tuberculosis *H37Rv; the remaining 6 epitopes (epitope IDs 10841, 18898, 103227, 103365, 103462, and 103503) presented specific mutations in BCGs exclusively. We infer that mutations in the 9 epitopes are conserved in both BCGs and* M. bovis,* while the other 6 also likely resulted from the originally attenuated BCG strains.

A total of 26 epitopes were classified as Group 3, involving 6 genes according to* M. tuberculosis* H37Rv (*Rv1582c*,* Rv2351c*,* Rv3874*,* Rv3875*,* Rv3878, *and* Rv3879c*). The* Rv1582c* gene, located in Regions of Differences 3 (RD3), encodes the probable phage protein PhiRv1. The* Rv2351c* gene, located in RD7, encodes MTP40 antigen. The remaining 4 genes (*Rv3874-3875* and* Rv3878-3879c*) were located in RD1 and encode CFP-10, ESAT-6, EspJ, and EspK, respectively [23]. It is known that RD1, RD3, and RD7 are missing in BCGs [28–30]. Therefore, the 26 epitopes had been lost before dissemination of the BCG ancestor.

The epitopes in Group 4 were involved in 4 genes, including* Rv1979c*,* Rv1980c*,* Rv1984c,* and* Rv1986*. These genes were found in RD2 by comparative genomics of BCGs and lost in BCG-Danish, BCG-Prague, BCG-Glaxo, BCG-Frappier, BCG-Phipps, BCG-Tice, and BCG-Pasteur, between 1927 and 1931 [28]. We experimentally verified the deletion of these 4 genes encoding 13 epitopes in BCG-Mexico.

Group 5 covered 23 epitopes with unique performances in different BCGs.

**Figure 3:**
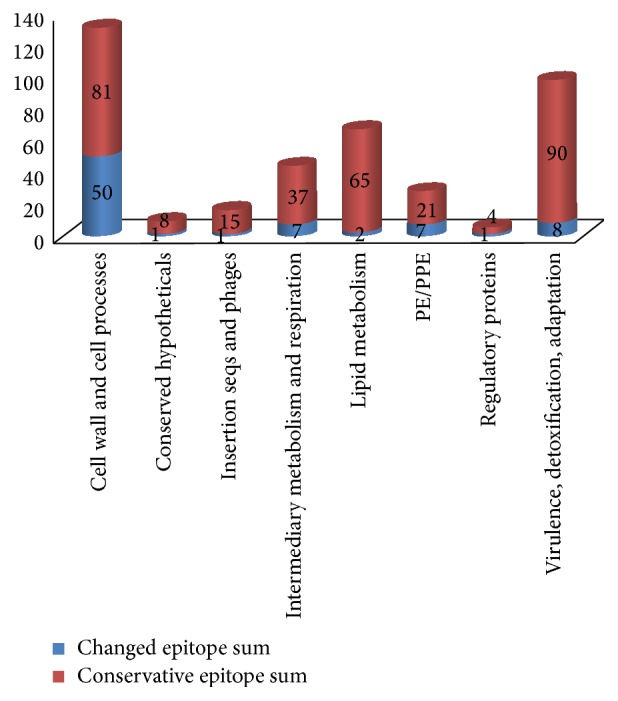
Variability of B cell epitopes in different functional categories.

In summary, the 398 epitopes could be divided into 2 categories, conserved and changed epitopes, based on nucleotide changes. The conserved epitopes contained 321 (80.7%) epitopes of Group 1. The changed ones included 77 (19.3%) epitopes of Groups 2, 3, 4, and 5. The epitopes encoding genes for “cell wall and cell processes” presented high variability (Figure 3).

### 3.4. Hyperconserved BCG Strains

**Figure 4:**
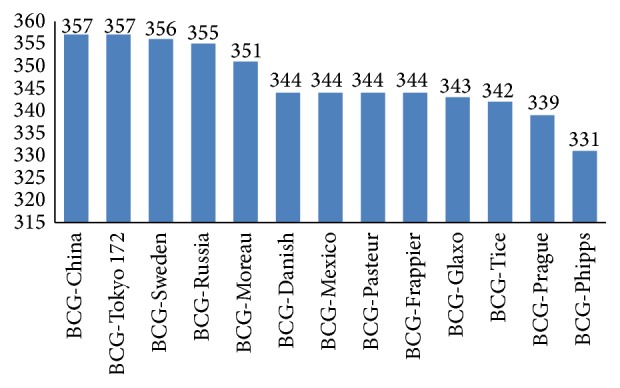
B cell epitope numbers in BCGs.

Here we compared B cell epitopes in 13 BCGs. All conserved and changed epitopes were analyzed according to BCG strain genomes. Finally, BCG-Tokyo 172 and BCG-China had the highest number of conserved epitopes (357 (89.7%)) (Figure 4).

## 4. Discussion

Tuberculosis, a horrible disease, had plagued humankind throughout the history. According to WHO data, 9.6 million new TB cases were estimated as well as 1.5 million TB related deaths in the world in 2014. BCG remains the only available and widely used vaccine against TB, although its protective effect against TB is not optimal, even with no protection for adults, because new TB vaccines are continually declared to be more effective in TB prevention. To date, at least 3 billion individuals have been immunized with BCG, and over 100 million doses of BCG are administered annually [31]. However, the variable protective efficacy of BCG in different populations is controversial [5, 32, 33]. Surprisingly, the immune mechanisms and protective differences of BCGs in populations remain poorly understood. Though much evidence presents T lymphocyte function in the adaptive immune response against tuberculosis, more and more findings have also demonstrated that B cells may play an important role in regulating the host response to TB invasion [10, 34].

This is the first study analyzing the protective differences of BCGs based on B cell epitopes distribution. It has been acknowledged that BCG is not a single strain and has lots of descendants worldwide. After the original BCG was disseminated around the world, it likely to undergo various selective pressures. As a result, different daughter-BCGs were produced, with variable protective efficacy. The BCG ancestor was serially cultured for at least 230 passages from 1908 to 1921, losing some important epitopes (Group 3, coding with RD1, RD3, and RD7); meanwhile, some point-mutations occurred in the epitopes (Group 2). Therefore, all 13 BCGs contained epitopes of Groups 1 and 2, losing those of Group 3. Then, some BCGs successfully retained Group 4 epitopes, including BCG-Moreau, BCG-Sweden, BCG-China, BCG-Russia, and BCG-Tokyo 172. Of these strains, BCG-Moreau, BCG-Sweden, and BCG-Russia produced more changes in the next few years. In addition, other daughter-BCGs underwent a period of continuous change, losing Group 4 epitopes (coding with RD2), including BCG-Prague, BCG-Phipps, BCG-Glaxo, BCG-Frappier, BCG-Danish, BCG-Tice, BCG-Mexico, and BCG-Pasteur. Of these strains, 4 BCGs (BCG-Prague, BCG-Phipps, BCG-Glaxo, and BCG-Tice) produced more change in B cell epitopes (Group 5) when they were disseminated to various parts of the world. The currently available BCGs exhibited distinct process of attenuation after 1924 [14].

Although the reasons for variable protective efficacy of BCGs are not fully understood, a key factor might be genetic differences in BCGs [14]. BCG-China and BCG-Tokyo 172 possessed the most B cell epitopes; these findings indicated that these 2 vaccines may conduct greater humoral immune response than the other BCGs. Studies have demonstrated that BCG-Tokyo 172, with the most experimentally verified T cell epitopes shown in our previous study [15], could evoke higher immune reaction than BCG-Danish and BCG-Russia [15, 35, 36]. And BCG-China had fewer verified T cell epitopes. Here we supposed that the BCG-China and BCG-Tokyo 172 could yield greater efficacy; in particular the BCG-Tokyo 172 could be the most efficient BCGs to date based on the analysis of verified B/T cell epitopes.

B cell epitope is the region of a pathogen that interacts with the host immune system. As shown above, most epitopes identified from genes encoding antigens on the pathogen surface were recognized by the host. Generally, antigen epitopes tend to be hypervariable to evade immune detection by the host [37, 38]. According to the above results, in spite of high variability of epitopes for “cell wall and cell processes,” most B cell epitopes (336 epitopes, Groups 1 and 2) were conserved in all BCGs without point-mutation in the coding region. This was consistent with the existence of T cell epitopes in BCGs and* M. tuberculosis* [15, 39] and inconsistent with a classical model of an evolutionary arms-race [40].

The BCG vaccine, an attenuated strain of* M. bovis*, mimics* M. tuberculosis* to evoke immune protection in the host. If the BCG becomes too attenuated to imitate the natural pathogen, it would not produce sufficient protection. We showed that BCG-China and BCG-Tokyo 172, containing the most B cell epitopes also had 41 altered B cell epitopes compared with* M. tuberculosis *H37Rv. Some important antigens of* M. tuberculosis* are therefore lost in all BCGs, such as proteins encoded with RD1, RD3, and RD7. In other words the BCG which has all necessary epitopes compared with natural pathogen would be the best attenuated vaccine against TB. Thus, rebuilding the BCG to recover more lost epitopes and enhance innate properties could improve its protective efficacy [25].

## 5. Conclusion

In this study, a total of 398 experimentally verified human B cell epitopes were analyzed with 13 BCG genomes. Of these, 321 were conserved in all BCGs; the remaining 77 B cell epitopes, encoding Regions of Differences or other important genes, have been altered during the ongoing dissemination. Our analysis, based on B cell epitopes distribution, also revealed that BCG-China especially BCG-Tokyo 172 could be the most effective BCG vaccines used in the world. Restoration of important antigens in BCG or enhancing effective B cell epitopes in vaccines could be a useful strategy for vaccine development.

## Supplementary Material

Table S1: 15 genome sequences analyzed in this study. 15 strains used in this paper including 13 BCGs, 1 *Mycobacterium tuberculosis* and 1 *Mycobacterium bovis*.
Table S2: 399 B cell epitopes and coding genes. 81 genes encoding 399 verified B cell epitopes according the genomes of *Mycobacterium tuberculosis* H37Rv and *Mycobacterium bovis* AF2122/97, respectively.
Table S3: Functional classification of genes coding B cell epitope. 81 genes which encoded B cell epitopes could be classified into 9 categories.
Table S4: B cell epitopes grouping in BCGs. All epitopes could be classified as 5 Groups based on distribution among BCGs.
