# Supplementary material for: Comparative Analysis of Human B Cell Epitopes Based on BCG Genomes
Source: Biomed Res Int. 2016 Jun 12;2016:3620141. doi: 10.1155/2016/3620141 (PMC4921132; doi:10.1155/2016/3620141)
Supplement: Supplementary file 1 — Table S1: 15 genome sequences analyzed in this study. 15 strains used in this paper including 13 BCGs, 1 Mycobacterium tuberculosis and 1 Mycobacterium bovis. Table S2: 399 B cell epitopes and coding genes. 81 genes encoding 399 verified B cell epitopes according the genomes of Mycobacterium tuberculosis H37Rv and Mycobacterium bovis AF2122/97, respectively. Table S3: Functional classification of genes coding B cell epitope. 81 genes which encoded B cell epitopes could be classified into 9 categories. Table S4: B cell epitopes grouping in BCGs. All epitopes could be classified as 5 Groups based on distribution among BCGs. [file 3620141.f1.pdf]

Table S1 15 genome sequences analyzed in this study

Strain	Size (Mb)	GC%	Accession	Reference
BCG-Moreau	4.16	65.4	AKYS00000000	16
BCG-Phipps	4.14	65.4	AKYT00000000	16
BCG-Sweden	4.09	65.3	AKYV00000000	16
BCG-Prague	4.13	65.3	AKYU00000000	16
BCG-Glaxo	4.21	65.4	AKYR00000000	16
BCG-Frappier	4.24	65.5	AKYQ00000000	16
BCG-China	4.18	65.3	AEZE00000000	17
BCG-Danish	4.17	65.3	AEZF00000000	17
BCG-Russia	4.18	65.3	AEZG00000000	17
BCG-Tice	4.17	65.3	AEZH00000000	17
BCG-Mexico	4.35	65.7	NC_016804	18
BCG-Pasteur	4.37	65.6	NC_008769	19
BCG-Tokyo 172	4.37	65.6	NC_012207	20
Mycobacterium tuberculosis H37Rv	4.41	65.6	NC_000962	21
Mycobacterium bovis AF2122/97	4.35	65.6	NC_002945	22

Table S2 399 B cell epitopes and coding genes

No.	Epitope ID	Coding Gene according to H37Rv	Coding Gene according to AF2122/97 gene	Epitope linear Sequence
1	103203	*Rv0066c*	*Mb0067c*	GELDNRGSQFYLAMY
2	103328	*Rv0066c*	*Mb0067c*	KTLDAAIGKLLDNDK
3	103550	*Rv0066c*	*Mb0067c*	RIFYKDAFAKHQELF
4	103745	*Rv0066c*	*Mb0067c*	YEEQMQDAFETGVMF
5	103113	*Rv0125*	*Mb0130*	DRFADFPALPLDPSA
6	103680	*Rv0125*	*Mb0130*	TWQTKSGGTRTGNVT
7	103040	*Rv0169*	*Mb0175*	ALTGLGDKFGESIVN
8	103544	*Rv0251c*	*Mb0257c*	RGEHRDEHTQDAGDK
9	103580	*Rv0287*	*Mb0295*	SAFAAKAGLMRHTIG
10	38988	*Rv0288*	*Mb0296*	LQSLGAEIAVEQAAL
11	103042	*Rv0288*	*Mb0296*	AMEDLVRAYHAMSST
12	103043	*Rv0288*	*Mb0296*	AMMARDTAEAAKWGG
13	41952	*Rv0315*	*Mb0323*	MLGTGTPNRARINFNC
14	53107	*Rv0350*	*Mb0358*	RAGRCPPR
15	53303	*Rv0350*	*Mb0358*	RCPPRRRA
16	73020	*Rv0350*	*Mb0358*	WRCPPRRR
17	48905	*Rv0350*	*Mb0358*	PPRRRAGR
18	6825	*Rv0350*	*Mb0358*	CPPRRRAG
19	49246	*Rv0350*	*Mb0358*	PRRRAGRC
20	55523	*Rv0350*	*Mb0358*	RRAGRCPP
21	55706	*Rv0350*	*Mb0358*	RRRAGRCP

22	63850	*Rv0350*	*Mb0358*	TGHWRCPP
23	25138	*Rv0350*	*Mb0358*	HWRCPPRR
24	61020	*Rv0350*	*Mb0358*	SSGCVTGH
25	20198	*Rv0350*	*Mb0358*	GHWRCPPR
26	61340	*Rv0350*	*Mb0358*	SSSGCVTG
27	55967	*Rv0350*	*Mb0358*	RSSSGCVT
28	71325	*Rv0350*	*Mb0358*	VTGHWRCP
29	7311	*Rv0350*	*Mb0358*	CVTGHWRC
30	819	*Rv0350*	*Mb0358*	AEAEGGTW
31	11021	*Rv0350*	*Mb0358*	EAEGGTWR
32	948	*Rv0350*	*Mb0358*	AEGGTWRI
33	1634	*Rv0350*	*Mb0358*	AGPGVAGS
34	1676	*Rv0350*	*Mb0358*	AGSGASDL
35	4364	*Rv0350*	*Mb0358*	ASDLRSSS
36	7474	*Rv0350*	*Mb0358*	DAAVAEAE
37	9268	*Rv0350*	*Mb0358*	DLRSSSGC
38	10406	*Rv0350*	*Mb0358*	DTLNKVDA
39	11057	*Rv0350*	*Mb0358*	EAGPGVAG
40	11451	*Rv0350*	*Mb0358*	EDTLNKVD
41	12182	*Rv0350*	*Mb0358*	EGGTWRIG
42	12688	*Rv0350*	*Mb0358*	EKFVKEQR
43	15991	*Rv0350*	*Mb0358*	FGHQVGDG
44	18764	*Rv0350*	*Mb0358*	GASDLRSS
45	19202	*Rv0350*	*Mb0358*	GEAGPGVA
46	20060	*Rv0350*	*Mb0358*	GGTWRIGY
47	20174	*Rv0350*	*Mb0358*	GHQVGDGE

48	21653	*Rv0350*	*Mb0358*	GPGVAGSG
49	22332	*Rv0350*	*Mb0358*	GSGASDLR
50	22398	*Rv0350*	*Mb0358*	GSKVPEDT
51	22912	*Rv0350*	*Mb0358*	GVAGSGAS
52	23337	*Rv0350*	*Mb0358*	GYFGHQVG
53	24658	*Rv0350*	*Mb0358*	HQVGDGEA
54	26348	*Rv0350*	*Mb0358*	IGYFGHQV
55	30877	*Rv0350*	*Mb0358*	KFVKEQRE
56	34154	*Rv0350*	*Mb0358*	KVPEDTLN
57	38259	*Rv0350*	*Mb0358*	LNKVDAAV
58	39216	*Rv0350*	*Mb0358*	LRSSSGCV
59	40649	*Rv0350*	*Mb0358*	LVYQTEKF
60	44613	*Rv0350*	*Mb0358*	NKVDAAVA
61	47260	*Rv0350*	*Mb0358*	PEDTLNKV
62	47769	*Rv0350*	*Mb0358*	PGVAGSGA
63	52501	*Rv0350*	*Mb0358*	QTEKFVKE
64	52698	*Rv0350*	*Mb0358*	QVGDGEAG
65	54157	*Rv0350*	*Mb0358*	RIGYFGHQ
66	57244	*Rv0350*	*Mb0358*	SDLRSSSG
67	57981	*Rv0350*	*Mb0358*	SGASDLRS
68	58963	*Rv0350*	*Mb0358*	SKVPEDTL
69	63420	*Rv0350*	*Mb0358*	TEKFVKEQ
70	64980	*Rv0350*	*Mb0358*	TLNKVDAA
71	67616	*Rv0350*	*Mb0358*	VAGSGASD
72	68636	*Rv0350*	*Mb0358*	VGDGEAGP
73	70277	*Rv0350*	*Mb0358*	VPEDTLNK

74	72142	*Rv0350*	*Mb0358*	VYQTEKFV
75	73033	*Rv0350*	*Mb0358*	WRIGYFGH
76	73817	*Rv0350*	*Mb0358*	YFGHQVGD
77	75560	*Rv0350*	*Mb0358*	YQTEKFVK
78	120639	*Rv0418*	*Mb0426*	IPVVSVTKSVGFQLRGQSGPTTVK
79	120810	*Rv0418*	*Mb0426*	MVNKSRMMPAV
80	9278	*Rv0440*	*Mb0448*	DLSLLGKARKVVVTKD
81	103346	*Rv0440*	*Mb0448*	LEPGVVAEKVRNLPA
82	103378	*Rv0440*	*Mb0448*	LPLLEKVIGAGKPLL
83	103554	*Rv0440*	*Mb0448*	RKVVVTKDETTIVEG
84	103605	*Rv0440*	*Mb0448*	SKVSTVKDLLPLLEK
85	103744	*Rv0440*	*Mb0448*	YDREKLQERLAKLAG
86	132976	*Rv0440*	*Mb0448*	AASIAGLFL
87	132994	*Rv0440*	*Mb0448*	ALVREGLRNVAAG
88	133264	*Rv0440*	*Mb0448*	NTFGLQLEL
89	175506	*Rv0440*	*Mb0448*	TLVVNKIRGTFKSVAVKAPG
90	103143	*Rv0447c*	*Mb0455c*	EMIEAVGYRSWPRYF
91	103264	*Rv0447c*	*Mb0455c*	HWLRPITPTFRPSWP
92	103431	*Rv0447c*	*Mb0455c*	MWELYLAYSEAGFRS
93	94762	*Rv0475*	*Mb0485*	AAPAKKAAPAKKAAA + METH(K179)
94	94763	*Rv0475*	*Mb0485*	AAPAKKAAPAKKAAA + METH(K179, K180)
95	122097	*Rv0538*	*Mb0552*	FPGWQPGMPTIPTAPPTTPV
96	122140	*Rv0538*	*Mb0552*	LQDSGVHDVAVISEAQAATA
97	122143	*Rv0538*	*Mb0552*	LSVVGDPDAPPTMVAVAPVA
98	122162	*Rv0538*	*Mb0552*	PIPVPIIIPPFPGWQPGMPT
99	103141	*Rv0652*	*Mb0671*	EKVAKEAADEAKAKL

100	103165	*Rv0652*	*Mb0671*	FEETFEVTAAAPVAV
101	103344	*Rv0652*	*Mb0671*	LEAAGDKKIGVIKVV
102	103426	*Rv0652*	*Mb0671*	MTLLELSDFVKKFEE
103	103589	*Rv0652*	*Mb0671*	SEFDVILEAAGDKKI
104	103301	*Rv0667*	*Mb0686*	KAMLFDGRSGEPFPY
105	103209	*Rv0667*	*Mb0686*	GFSEIMRSTLEKDNT
106	103255	*Rv0667*	*Mb0686*	HGTCANQCPIVDAGD
107	103361	*Rv0667*	*Mb0686*	LLDIYRKLRPGEPPT
108	103669	*Rv0667*	*Mb0686*	TSEQIVERFGFSEIM
109	103716	*Rv0667*	*Mb0686*	VRERMTTQDVEAITP
110	103037	*Rv0668*	*Mb0687*	AKVRRERMGHIELAA
111	103078	*Rv0668*	*Mb0687*	AYVITSVDEEMRHNE
112	103244	*Rv0668*	*Mb0687*	GVPSRLGYLLDLAPK
113	103464	*Rv0668*	*Mb0687*	PETINYRTLKPEKDG
114	121059	*Rv0755c*	*Mb0777c*	VVHPAVVQANRVRTWLLAVSNVFGQ
115	103198	*Rv0916c*	*Mb0940c*	GDLPTIGTAVSARNT
116	103420	*Rv0916c*	*Mb0940c*	MSFVTIQPVVLAAAT
117	103055	*Rv0928*	*Mb0951*	AQAQHLTMANIVTSA
118	103153	*Rv0928*	*Mb0951*	ESGTTDNFQRYLQAA
119	103531	*Rv0928*	*Mb0951*	QSGLGDNGYIPIPDE
120	103577	*Rv0928*	*Mb0951*	SAAAKNTPGSITYNE
121	103536	*Rv0931c*	*Mb0955c*	QVGSQFGPYQLLRLL
122	103754	*Rv0931c*	*Mb0955c*	YRADSVERLIAAHLM
123	103579	*Rv0932c*	*Mb0956c*	SAERCGSPAWDLPTV
124	103462	*Rv0932c*	*Mb0956c*	PDRSAERCGSPAWDL
125	103010	*Rv0932c*	*Mb0956c*	ACGGGTNSSSSGAGG

126	103230	*Rv0932c*	*Mb0956c*	GPGQEGLDQYGSIPL
127	103493	*Rv0932c*	*Mb0956c*	PPTPISVIFRSDKSG
128	103569	*Rv0932c*	*Mb0956c*	RSCPGYTLDYNANGS
129	103738	*Rv0932c*	*Mb0956c*	WSFAVGKQLNMAQII
130	103751	*Rv0932c*	*Mb0956c*	YNANGSGAGVTQFLN
131	103061	*Rv0934*	*Mb0959*	ASFLDQVHFQPLPPA
132	103227	*Rv0934*	*Mb0959*	GNKASFLDQVHFQPL
133	9924	*Rv0934*	*Mb0959*	DQVHFQPLPPAVVKLSDALI
134	103633	*Rv0934*	*Mb0959*	TAQTLQAFLHWAITD
135	7472	*Rv0934*	*Mb0959*	DAATAQTLQAFLHWAITDGN
136	103659	*Rv0934*	*Mb0959*	TLQAFLHWAITDGNK
137	120481	*Rv0934*	*Mb0959*	EHLKLNGKVLAAMYQ
138	103373	*Rv0934*	*Mb0959*	LPGVSEHLKLNGKVL
139	103004	*Rv0934*	*Mb0959*	AAHKGLMNIALAISA
140	103018	*Rv0934*	*Mb0959*	AFLHWAITDGNKASF
141	103021	*Rv0934*	*Mb0959*	AGAGTVATTPASSPV
142	103029	*Rv0934*	*Mb0959*	AGTVNIGASDAYLSE
143	103090	*Rv0934*	*Mb0959*	CVAYIGISFLDQASQ
144	103105	*Rv0934*	*Mb0959*	DGYPIINYEYAIVNN
145	103137	*Rv0934*	*Mb0959*	EGWGKSPGFGTTVDF
146	103260	*Rv0934*	*Mb0959*	HRSDGSGDTFLFTQY
147	103286	*Rv0934*	*Mb0959*	ISMIDGPAPDGYPII
148	103304	*Rv0934*	*Mb0959*	KGLMNIALAISAQQV
149	103349	*Rv0934*	*Mb0959*	LGENGNGGMVTGCAE
150	103386	*Rv0934*	*Mb0959*	LSEGDMAAHKGLMNI
151	103415	*Rv0934*	*Mb0959*	MNIALAISAQQVNYN

152	103516	*Rv0934*	*Mb0959*	QDPEGWGKSPGFGTT
153	103547	*Rv0934*	*Mb0959*	RGLGEAQLGNSSGNF
154	103747	*Rv0934*	*Mb0959*	YIGISFLDQASQRGL
155	120511	*Rv0956*	*Mb0981*	ETLHERIKVTERRLLVAAVAALATH
156	120410	*Rv0956*	*Mb0981*	ARLVVLASGTGSLLR
157	120537	*Rv1114*	*Mb1144*	FPVVTHDEVLRLVGRRRLWGRG
158	103365	*Rv1196*	*Mb1228*	LMILIATNLLGQNTP
159	103503	*Rv1196*	*Mb1228*	PVIAENRAELMILIA
160	103007	*Rv1196*	*Mb1228*	AAQAVQTAAQNGVRA
161	103452	*Rv1196*	*Mb1228*	NTPAIAVNEAEYGEM
162	120569	*Rv1382*	*Mb1417*	GSLIFAAVLVMLIAVLARLMMRGWR
163	120912	*Rv1382*	*Mb1417*	RGVAGKVVAGIGILAIRWRLPSGT
164	120802	*Rv1434*	*Mb1469*	MRASPAERVDGAYAGAGPHTQSV
165	120399	*Rv1582c*	*Mb1608c*	AIWRRIRVVPFEVVIPADEQD
166	103274	*Rv1636*	*Mb1662*	ILKDESYKVTGTAPI
167	103620	*Rv1636*	*Mb1662*	SVPANVSRRAKVDVL
168	103694	*Rv1636*	*Mb1662*	VGNVGLSTIAGRLLG
169	103425	*Rv1690*	*Mb1716*	MTAHTHDGTRTWRTG
170	103434	*Rv1690*	*Mb1716*	NAFLTALTNAGIAYD
171	103693	*Rv1690*	*Mb1716*	VFGGAASCAAPIQAD
172	103128	*Rv1793*	*Mb1821*	EAEHQAIVRDVLAAG
173	103170	*Rv1793*	*Mb1821*	FGDVDAHGAMIRAQA
174	103121	*Rv1827*	*Mb1858*	DVGSLNGTYVNREPV
175	103291	*Rv1827*	*Mb1858*	ITSAGRHPDSDIFLD
176	103347	*Rv1837c*	*Mb1868c*	LFSRVEDVLGLPQNT
177	103636	*Rv1837c*	*Mb1868c*	TCELFSRVEDVLGLP

178	103641	*Rv1837c*	*Mb1868c*	TDVIPETDGAEKGPT
179	103651	*Rv1837c*	*Mb1868c*	TIEQLLTIPLAKELA
180	72741	*Rv1862*	*Mb1893*	WLKLGLVEFGGVAKLNAEVMS
181	120392	*Rv1866*	*Mb1897*	AAVQAAWAVLVAYFNRLRCGTGDY
182	120452	*Rv1866*	*Mb1897*	DRYRHLVALSITDFGAAGPRSSWRA
183	121017	*Rv1866*	*Mb1897*	TTIFPDHVVGRVGALLALAALIHRD
184	18898	*Rv1886c*	*Mb1918c*	GCQTYKWETFLTSELPQWLS
185	10841	*Rv1886c*	*Mb1918c*	DWYSPACGKAGCQTYKWETF
186	223	*Rv1886c*	*Mb1918c*	AAIGLSMAGSSAMILAAYHP
187	503	*Rv1886c*	*Mb1918c*	AAVVLPGLVGLAGGAATAGA
188	1545	*Rv1886c*	*Mb1918c*	AGGYKAADMWGPSSDPAWER
189	2695	*Rv1886c*	*Mb1918c*	ALLDPSQGMGPSLIGLAMGD
190	3400	*Rv1886c*	*Mb1918c*	ANRAVKPTGSAAIGLSMAGS
191	6323	*Rv1886c*	*Mb1918c*	CGNGTPNELGGANIPAEFLE
192	8688	*Rv1886c*	*Mb1918c*	DIKVQFQSGGNNSPAVYLLD
193	18700	*Rv1886c*	*Mb1918c*	GANIPAEFLENFVRSSNLKF
194	21096	*Rv1886c*	*Mb1918c*	GLRAQDDYNGWDINTPAFEW
195	21797	*Rv1886c*	*Mb1918c*	GPSSDPAWERNDPTQQIPKL
196	34776	*Rv1886c*	*Mb1918c*	LAGGAATAGAFSRPGLPVEY
197	40165	*Rv1886c*	*Mb1918c*	LTSELPQWLSANRAVKPTGS
198	42790	*Rv1886c*	*Mb1918c*	MTDVSRKIRAWGRRLMIGTA
199	43332	*Rv1886c*	*Mb1918c*	NAVFNFPPNGTHSWEYWGAQ
200	43514	*Rv1886c*	*Mb1918c*	NDPTQQIPKLVANNTRLWVY
201	45250	*Rv1886c*	*Mb1918c*	NNSPAVYLLDGLRAQDDYNG
202	48646	*Rv1886c*	*Mb1918c*	PNGTHSWEYWGAQ
203	49421	*Rv1886c*	*Mb1918c*	PSLIGLAMGDAGGYKAADMW

204	49872	*Rv1886c*	*Mb1918c*	PVGGQSSFYSDWYSPACGKA
205	50442	*Rv1886c*	*Mb1918c*	QDAYNAAGGHNAVFNFPPNG
206	52026	*Rv1886c*	*Mb1918c*	QQFIYAGSLSALLDPSQGMG
207	56895	*Rv1886c*	*Mb1918c*	SAMILAAYHPQQFIYAGSLS
208	64079	*Rv1886c*	*Mb1918c*	THSWEYWGAQLNAMKGDLQS
209	67697	*Rv1886c*	*Mb1918c*	VANNTRLWVYCGNGTPNELG
210	72314	*Rv1886c*	*Mb1918c*	WDINTPAFEWYYQSGLSIVM
211	72515	*Rv1886c*	*Mb1918c*	WGRRLMIGTAAAVVLPGLVG
212	76584	*Rv1886c*	*Mb1918c*	YYQSGLSIVMPVGGQSSFYS
213	103272	*Rv1886c*	*Mb1918c*	IGLSMAGSSAMILAA
214	103457	*Rv1886c*	*Mb1918c*	PAEFLENFVRSSNLK
215	103530	*Rv1886c*	*Mb1918c*	QSGGNNSPAVYLLDG
216	103532	*Rv1886c*	*Mb1918c*	QSSFYSDWYSPACGK
217	103578	*Rv1886c*	*Mb1918c*	SAAIGLSMAGSSAMI
218	103668	*Rv1886c*	*Mb1918c*	TSELPQWLSANRAVK
219	103729	*Rv1886c*	*Mb1918c*	WGPSSDPAWERNDPT
220	103732	*Rv1886c*	*Mb1918c*	WLSANRAVKPTGSAA
221	103067	*Rv1926c*	*Mb1961c*	ATIPQGEQSTGKIYF
222	103283	*Rv1926c*	*Mb1961c*	IRGSVTPAVSQFNAR
223	103355	*Rv1926c*	*Mb1961c*	LGWKVSDLKSSTAVI
224	103479	*Rv1926c*	*Mb1961c*	PGYPVAGQVWEATAT
225	103501	*Rv1926c*	*Mb1961c*	PVAGQVWEATATVNA
226	103537	*Rv1926c*	*Mb1961c*	QVVLGWKVSDLKSST
227	120441	*Rv1979c*	*Mb2001c*	DKVLPLVAIVVSVGLAVSYD
228	120788	*Rv1979c*	*Mb2001c*	LTFLGFIGVLLAINLFGNRAIKWAN
229	22997	*Rv1980c*	*Mb2002c*	GVIFFFNPGELLPEA

230	24522	*Rv1980c*	*Mb2002c*	HPTTTYKAFDWDQAYRKPIT
231	44381	*Rv1980c*	*Mb2002c*	NITSATYQSAIPPRGTQAVV
232	103145	*Rv1980c*	*Mb2002c*	ENYIAQTRDKFLSAA
233	103525	*Rv1980c*	*Mb2002c*	QNAGGTHPTTTYKAF
234	103635	*Rv1980c*	*Mb2002c*	TAVVLLCCSGVATAA
235	103661	*Rv1980c*	*Mb2002c*	TNDGVIFFFNPGELL
236	103327	*Rv1984c*	*Mb2006c*	KTINLCAPDDPICTG
237	103348	*Rv1984c*	*Mb2006c*	LGDVGEAFVDSLTSQ
238	103686	*Rv1984c*	*Mb2006c*	VALFGEPSSGFSSML
239	120777	*Rv1986*	*Mb2008*	LPVVALCTVSDIVLIAAGIAGFGA
240	20662	*Rv2031c*	*Mb2057c*	GKPTEKHIQIRSTN
241	53355	*Rv2031c*	*Mb2057c*	RDGQLTIKAERTEQKDFDGR
242	56043	*Rv2031c*	*Mb2057c*	RTEQKDFDGRSEFAYGSFVR
243	67156	*Rv2031c*	*Mb2057c*	TVSLPVGADEDDIKATYDKG
244	103130	*Rv2031c*	*Mb2057c*	EDEMKEGRYEVRAEL
245	103180	*Rv2031c*	*Mb2057c*	FPSFAGLRPTFDTRL
246	103718	*Rv2031c*	*Mb2057c*	VSVAVSEGKPTEKHI
247	103266	*Rv2185c*	*Mb2207c*	IADIEAYPQWISEYK
248	103731	*Rv2185c*	*Mb2207c*	WISEYKEVEILEADD
249	103296	*Rv2246*	*Mb2270*	IVMSTNNDDPAGACR
250	120892	*Rv2346c*	*Mb2375c*	QKVQAAGNNMAQT
251	21765	*Rv2351c*	*no existed*	GPRLYGEMTMQGTRKPRPSGP
252	27635	*Rv2351c*	*no existed*	INFNCEVWSNVSETISGPRLY
253	41948	*Rv2351c*	*no existed*	MLGNAPSVVPNTTLGM
254	66693	*Rv2351c*	*no existed*	TTLGMHCGSFGSAPSNG
255	4556	*Rv2351c*	*no existed*	ASMLGTVTNSPGVPAVPWGA

256	103737	*Rv2430c*	*Mb2456c*	WRSLDVEMTAVQRSF
257	103338	*Rv2453c*	*Mb2480c*	LAPDTVPLAGVVLAG
258	103472	*Rv2453c*	*Mb2480c*	PGGTTTLVEHMVGIL
259	103548	*Rv2453c*	*Mb2480c*	RGLRAAAEAGVRLAF
260	103687	*Rv2453c*	*Mb2480c*	VCAVDMPYLTVELIE
261	9474	*Rv2608*	*Mb2640*	DNIGNANIGFGN
262	9475	*Rv2608*	*Mb2640*	DNIGNANIGFGNRGDANIGIGNIGDRNLGIGNTGNW
263	10022	*Rv2608*	*Mb2640*	DRNLGIGNTGNW
264	20281	*Rv2608*	*Mb2640*	GIGNIGDRNLGI
265	21497	*Rv2608*	*Mb2640*	GNPNRPDGGILT
266	39354	*Rv2608*	*Mb2640*	LSFTLTGNPNRP
267	44231	*Rv2608*	*Mb2640*	NIGFGNRGDANI
268	53833	*Rv2608*	*Mb2640*	RGDANIGIGNIG
269	55157	*Rv2608*	*Mb2640*	RPGLDELSFTLT
270	55158	*Rv2608*	*Mb2640*	RPGLDELSFTLTGNPNRPDGGILT
271	103393	*Rv2608*	*Mb2640*	LTTYILLPSQDLPLL
272	103116	*Rv2626c*	*Mb2659c*	DSIYYVDANASIQEM
273	103197	*Rv2626c*	*Mb2659c*	GDDDRLHGMLTDRDI
274	103247	*Rv2629*	*Mb2662*	GYPVHKPVTAGWNGY
275	103162	*Rv2780*	*Mb2803*	FCGRIHTRYSSAYEL
276	103002	*Rv2873*	*Mb2898*	AAASLAAIAIAFLAG
277	103118	*Rv2873*	*Mb2898*	DTLNGGEYTVFAPTN
278	103262	*Rv2873*	*Mb2898*	HVIAGQASPSRIDGT
279	103267	*Rv2873*	*Mb2898*	IAFLAGCSSTKPVSQ
280	103000	*Rv2875*	*Mb2900*	AAAGDLVGPGCAEYA
281	103199	*Rv2875*	*Mb2900*	GDLVGPGCAEYAAAN

282	103720	*Rv2875*	*Mb2900*	VTVTGQGNSLKVGNA
283	103195	*Rv2875*	*Mb2900*	GASVTVTGQGNSLKV
284	103082	*Rv2875*	*Mb2900*	CGGVSTANATVYMID
285	103746	*Rv2875*	*Mb2900*	YHVVAGQTSPANVVG
286	103339	*Rv2940c*	*Mb2965c*	LASTLTDALKSHGPQ
287	103185	*Rv2940c*	*Mb2965c*	FRMELLSLPQDEWAG
288	103246	*Rv2940c*	*Mb2965c*	GYIPILGAPWLADLV
289	103384	*Rv2940c*	*Mb2965c*	LQAHPHAVNGTAMRE
290	103476	*Rv2940c*	*Mb2965c*	PGQGAYAAANSWVDV
291	103480	*Rv2940c*	*Mb2965c*	PILGAPWLADLVRRS
292	103490	*Rv2940c*	*Mb2965c*	PPGYDITALLQAHPH
293	103612	*Rv2940c*	*Mb2965c*	SPHPLLTHAVEQTGR
294	103629	*Rv2940c*	*Mb2965c*	TAALRAAEDDCPPPG
295	103700	*Rv2940c*	*Mb2965c*	VHNVAALPGAAYCEM
296	103125	*Rv2941*	*Mb2966*	DVVQHVARRPGESPP
297	103756	*Rv2941*	*Mb2966*	YTFKEDEYPSTAYLQ
298	103017	*Rv2957*	*Mb2981*	AFDLDRLLFKRNICH
299	103031	*Rv2958c*	*Mb2982c*	AIANAYWSPQARRRF
300	103179	*Rv2958c*	*Mb2982c*	FPHHPIHTVPSEEVL
301	103566	*Rv2958c*	*Mb2982c*	RRFPLPDVPWTRFFG
302	103677	*Rv2958c*	*Mb2982c*	TVIAATAGRNHLKNV
303	103703	*Rv2958c*	*Mb2982c*	VLCNGGSPTTQQALA
304	103112	*Rv2962c*	*Mb2986c*	DPRYNQLLGPLPFRH
305	103549	*Rv2962c*	*Mb2986c*	RHGLSSLGWNLCRIF
306	103717	*Rv2962c*	*Mb2986c*	VRRRHGLSSLGWNLC
307	103755	*Rv2962c*	*Mb2986c*	YRLERPLLFALQCMP

308	120587	*Rv2994*	*Mb3018*	HSLLWIGVFLFLGGMAAGGCNS
309	120630	*Rv2994*	*Mb3018*	IMIVSLGVTASSFLFINGVAFLIPR
310	120878	*Rv2994*	*Mb3018*	PSWGLVVTMFAWGYLLDHVGERMV
311	120961	*Rv2994*	*Mb3018*	SPYRGSSILWRIHAASALLMMP
312	121057	*Rv2994*	*Mb3018*	VTVTFMLVWLINHHGWSVAQ
313	103359	*Rv3133c*	*Mb3157c*	LILTSYTSDEAMLDA
314	120904	*Rv3219*	*Mb3245*	RALKRRNARTKAR
315	103403	*Rv3221c*	*Mb3247c*	MAEDVRAEIVASVLE
316	103708	*Rv3221c*	*Mb3247c*	VLLESMKMEIPVLAE
317	103102	*Rv3339c*	*Mb3371c*	DEMTRVIWKLIKDML
318	103297	*Rv3339c*	*Mb3371c*	IWKLIKDMLILPYLD
319	103432	*Rv3339c*	*Mb3371c*	MWLSPNGTIRNILGG
320	103352	*Rv3347c*	*Mb3380c*	LGGGVGAFLNALFAG
321	103038	*Rv3347c*	*Mb3380c*	ALMSGNFSNGILWRG
322	22387	*Rv3347c*	*Mb3379c*	GSINTGWFNTGNANTG
323	103022	*Rv3347c*	*Mb3380c*	AGFTAPATTLLSALG
324	103469	*Rv3347c*	*Mb3380c*	PFNVNLKLQFLHDAF
325	103645	*Rv3347c*	*Mb3379c*	TGGGGGSGFSNSGSG
326	19562	*Rv3347c*	*Mb3379c*	GFKVRPSFSFFAVGPDGMP
327	103482	*Rv3347c*	*Mb3380c*	PLDTPPAPAPPPFRL
328	103664	*Rv3347c*	*Mb3380c*	TPPAPAPPPFRLPLL
329	103534	*Rv3354*	*Mb3389*	QSVCPILAEPGGSFN
330	4132	*Rv3418c*	*Mb3452c*	ARDVLAVVSK
331	8900	*Rv3418c*	*Mb3452c*	DKILVQANEAETTTA
332	14574	*Rv3418c*	*Mb3452c*	ETTTASGLVIPDTAK
333	35316	*Rv3418c*	*Mb3452c*	LDVAEGDTVIYSKYG

334	41071	*Rv3418c*	*Mb3452c*	MAKVNIKPLEDKILV
335	47209	*Rv3418c*	*Mb3452c*	PDTAKEKPQEGTVVA
336	72294	*Rv3418c*	*Mb3452c*	WDEDGEKRIPLDVAE
337	103013	*Rv3418c*	*Mb3452c*	AEGDTVIYSKYGGTE
338	103695	*Rv3418c*	*Mb3452c*	VGPGRWDEDGEKRIP
339	120887	*Rv3736*	*Mb3763*	QDVGNYDAFISIRAAIRAIESA
340	433	*Rv3763*	*Mb3789*	AASGPKVVIDGKDQNVTGSV
341	4334	*Rv3763*	*Mb3789*	ASATKDGSHYKITGTATGVD
342	4990	*Rv3763*	*Mb3789*	ATGVDMANPMSPVNKSFEIE
343	8464	*Rv3763*	*Mb3789*	DGNPPEVKSVGLGNVNGVTL
344	25315	*Rv3763*	*Mb3789*	IAIGGAATGIAAVLTDGNPP
345	42633	*Rv3763*	*Mb3789*	MSPVNKSFEIEVTCS
346	58022	*Rv3763*	*Mb3789*	SGETTTAAGTTASPGAASGP
347	67607	*Rv3763*	*Mb3789*	VAGLSGCSSNKSTTGSGETT
348	71346	*Rv3763*	*Mb3789*	VTGSVVCTTAAGNVNIAIGG
349	105967	*Rv3763*	*Mb3789*	MKRGLTVAVAGAAILVAGLS
350	105976	*Rv3763*	*Mb3789*	NGVTLGYTSGTCQGNASATK
351	38439	*Rv3803c*	*Mb3833c*	LPDWLAANRGLAPGG
352	46806	*Rv3803c*	*Mb3833c*	PAGGAYSMYTNWEQD
353	1522	*Rv3804c*	*Mb3834c*	AGGGHNGVFDFPDSG
354	3402	*Rv3804c*	*Mb3834c*	ANRHVKPTGSAVVGL
355	17838	*Rv3804c*	*Mb3834c*	FSRPGLPVEYLQVPSPSMGR
356	39011	*Rv3804c*	*Mb3834c*	LQVPSPSMGRDIKVQFQSGG
357	49333	*Rv3804c*	*Mb3834c*	PSDLGGNNLPAKFLE
358	51790	*Rv3804c*	*Mb3834c*	QPACRKAGCQTYKWE
359	70010	*Rv3804c*	*Mb3834c*	VMPVGGQSSFYSDWY

360	98793	*Rv3841*	*Mb3871*	GEQFMQWFLQEQIEEVALMA
361	98906	*Rv3841*	*Mb3871*	MLVQHLLDRDLRVEIPGVDT
362	103697	*Rv3864*	*Mb3894*	VGQVTATMGQLQQLV
363	103176	*Rv3864*	*Mb3894*	FLGGFAGLPSLGFGN
364	103331	*Rv3864*	*Mb3894*	LAALPDFLGGFAGLP
365	103510	*Rv3864*	*Mb3894*	QAQLISSQAQQGGQQ
366	103245	*Rv3864*	*Mb3894*	GVYKVCKGLEKIPLL
367	103340	*Rv3864*	*Mb3894*	LCKTTSNFIWGQLLL
368	103649	*Rv3864*	*Mb3894*	TGNMISNQAKYVSDT
369	187	*Rv3874*	*Mb3904*	AAGTAAQAAVVRFQE
370	655	*Rv3874*	*Mb3904*	ADEEQQQALSSQMGF
371	3934	*Rv3874*	*Mb3904*	AQAAVVRFQEAANKQ
372	21422	*Rv3874*	*Mb3904*	GNFERISGDLKTQID
373	28505	*Rv3874*	*Mb3904*	ISGDLKTQIDQVEST
374	32971	*Rv3874*	*Mb3904*	KQELDEISTNIRQAG
375	33831	*Rv3874*	*Mb3904*	KTQIDQVESTAGSLQ
376	34927	*Rv3874*	*Mb3904*	LAQEAGNFERISGDL
377	37040	*Rv3874*	*Mb3904*	LKTQIDQVESTAG
378	41028	*Rv3874*	*Mb3904*	MAEMKTDAATLAQEA
379	50549	*Rv3874*	*Mb3904*	QEAGNFERISGDLKT
380	103519	*Rv3874*	*Mb3904*	QGQWRGAAGTAAQAA
381	120421	*Rv3874*	*Mb3904*	AVVRFQEAANKQK
382	60	*Rv3875*	*Mb3905*	AAASAIQGNVTSIHSL
383	35139	*Rv3875*	*Mb3905*	LDEGKQSLTKLAAAWG
384	46500	*Rv3875*	*Mb3905*	NVTSIHSLLDEGKQSL
385	52048	*Rv3875*	*Mb3905*	QQKWDATATELNN

386	61578	*Rv3875*	*Mb3905*	STEGNVTGMFA
387	120550	*Rv3878*	*Mb3908*	GDVQPAEVVAAAR
388	120870	*Rv3879c*	*Mb3909c*	PHVKPAALAEQPG
389	103371	*Rv3881c*	*Mb3911c*	LPADTAAQLTSAGRE
390	103208	*Rv3881c*	*Mb3911c*	GFLMPPSDGSGVTPG
391	103258	*Rv3881c*	*Mb3911c*	HQGQGGAKSKGSQQE
392	103511	*Rv3881c*	*Mb3911c*	QAQYVAQLHVWARRE
393	120408	*Rv3883c*	*Mb3913c*	APYNVRRLPPPVVEP
394	120428	*Rv3883c*	*Mb3913c*	DDLVGAGVIDAVA
395	98728	*Rv3914*	*Mb3945*	DFWATWCGPCKMVAPVLEEI
396	98784	*Rv3914*	*Mb3945*	GAKGKAALLRELSDVVPNLN
397	98852	*Rv3914*	*Mb3945*	KMVAPVLEEIATERATDLTV
398	103284	*Rv3922c*	*Mb3953c*	IRLAKCGPWHRGGWD
399	103385	*Rv3922c*	*Mb3953c*	LRPASCRFVPTCSQY

Table S3 Functional classification of genes coding B cell epitope

H37Rv gene	*M.bovis* Gene	Gene Category
Rv0287	*Mb0295*	cell wall and cell processes
Rv0288	*Mb0296*	cell wall and cell processes
Rv0418	*Mb0426*	cell wall and cell processes
Rv0475	*Mb0485*	cell wall and cell processes
Rv0538	*Mb0552*	cell wall and cell processes
Rv0928	*Mb0951*	cell wall and cell processes
Rv0932c	*Mb0956c*	cell wall and cell processes
Rv0934	*Mb0959*	cell wall and cell processes
Rv1382	*Mb1417*	cell wall and cell processes

Rv1690	*Mb1716*	cell wall and cell processes
Rv1793	*Mb1821*	cell wall and cell processes
Rv1926c	*Mb1961c*	cell wall and cell processes
Rv1979c	*Mb2001c*	cell wall and cell processes
Rv1980c	*Mb2002c*	cell wall and cell processes
Rv1984c	*Mb2006c*	cell wall and cell processes
Rv1986	*Mb2008*	cell wall and cell processes
Rv2346c	*Mb2375c*	cell wall and cell processes
Rv2873	*Mb2898*	cell wall and cell processes
Rv2875	*Mb2900*	cell wall and cell processes
Rv2994	*Mb3018*	cell wall and cell processes
Rv3763	*Mb3789*	cell wall and cell processes
Rv3864	*Mb3894*	cell wall and cell processes
Rv3874	*Mb3904*	cell wall and cell processes
Rv3875	*Mb3905*	cell wall and cell processes
Rv3878	*Mb3908*	cell wall and cell processes
Rv3879c	*Mb3909c*	cell wall and cell processes
Rv3881c	*Mb3911c*	cell wall and cell processes
Rv1434	*Mb1469*	conserved hypotheticals
Rv1827	*Mb1858*	conserved hypotheticals
Rv2185c	*Mb2207c*	conserved hypotheticals
Rv2626c	*Mb2659c*	conserved hypotheticals
Rv2629	*Mb2662*	conserved hypotheticals
Rv3354	*Mb3389*	conserved hypotheticals
Rv0652	*Mb0671*	information pathways
Rv0667	*Mb0686*	information pathways

Rv0668	*Mb0687*	information pathways
Rv1582c	*Mb1608c*	insertion seqs and phages
Rv0066c	*Mb0067c*	intermediary metabolism and respiration
Rv0125	*Mb0130*	intermediary metabolism and respiration
Rv0315	*Mb0323*	intermediary metabolism and respiration
Rv0956	*Mb0981*	intermediary metabolism and respiration
Rv1837c	*Mb1868c*	intermediary metabolism and respiration
Rv1862	*Mb1893*	intermediary metabolism and respiration
Rv2351c	*no existed*	intermediary metabolism and respiration
Rv2453c	*Mb2480c*	intermediary metabolism and respiration
Rv2780	*Mb2803*	intermediary metabolism and respiration
Rv2957	*Mb2981*	intermediary metabolism and respiration
Rv2958c	*Mb2982c*	intermediary metabolism and respiration
Rv2962c	*Mb2986c*	intermediary metabolism and respiration
Rv3339c	*Mb3371c*	intermediary metabolism and respiration
Rv3841	*Mb3871*	intermediary metabolism and respiration
Rv3883c	*Mb3913c*	intermediary metabolism and respiration
Rv3914	*Mb3945*	intermediary metabolism and respiration
Rv0447c	*Mb0455c*	lipid metabolism
Rv1866	*Mb1897*	lipid metabolism
Rv1886c	*Mb1918c*	lipid metabolism
Rv2246	*Mb2270*	lipid metabolism
Rv2940c	*Mb2965c*	lipid metabolism
Rv2941	*Mb2966*	lipid metabolism
Rv3221c	*Mb3247c*	lipid metabolism
Rv3803c	*Mb3833c*	lipid metabolism

Rv3804c	*Mb3834c*	lipid metabolism
Rv0755c	*Mb0777c*	PE/PPE
Rv0916c	*Mb0940c*	PE/PPE
Rv1196	*Mb1228*	PE/PPE
Rv2430c	*Mb2456c*	PE/PPE
Rv2608	*Mb2640*	PE/PPE
Rv3347c	*Mb3379c/Mb3380c*	PE/PPE
Rv0931c	*Mb0955c*	regulatory proteins
Rv3133c	*Mb3157c*	regulatory proteins
Rv3219	*Mb3245*	regulatory proteins
Rv3736	*Mb3763*	regulatory proteins
Rv0169	*Mb0175*	virulence, detoxification, adaptation
Rv0251c	*Mb0257c*	virulence, detoxification, adaptation
Rv0350	*Mb0358*	virulence, detoxification, adaptation
Rv0440	*Mb0448*	virulence, detoxification, adaptation
Rv1114	*Mb1144*	virulence, detoxification, adaptation
Rv1636	*Mb1662*	virulence, detoxification, adaptation
Rv2031c	*Mb2057c*	virulence, detoxification, adaptation
Rv3418c	*Mb3452c*	virulence, detoxification, adaptation
Rv3922c	*Mb3953c*	virulence, detoxification, adaptation

Table S4 B cell epitopes grouping in BCGs

Group	Epitope ID	Characteristic
1	103203	present in 13 BCGs
1	103328	present in 13 BCGs
1	103550	present in 13 BCGs

1	103745	present in 13 BCGs
1	103113	present in 13 BCGs
1	103680	present in 13 BCGs
1	103040	present in 13 BCGs
1	103544	present in 13 BCGs
1	103580	present in 13 BCGs
1	38988	present in 13 BCGs
1	103042	present in 13 BCGs
1	103043	present in 13 BCGs
1	819	present in 13 BCGs
1	11021	present in 13 BCGs
1	948	present in 13 BCGs
1	1634	present in 13 BCGs
1	1676	present in 13 BCGs
1	4364	present in 13 BCGs
1	7474	present in 13 BCGs
1	9268	present in 13 BCGs
1	10406	present in 13 BCGs
1	11057	present in 13 BCGs
1	11451	present in 13 BCGs
1	12182	present in 13 BCGs
1	12688	present in 13 BCGs
1	15991	present in 13 BCGs
1	18764	present in 13 BCGs
1	19202	present in 13 BCGs
1	20060	present in 13 BCGs

1	20174	present in 13 BCGs
1	21653	present in 13 BCGs
1	22332	present in 13 BCGs
1	22398	present in 13 BCGs
1	22912	present in 13 BCGs
1	23337	present in 13 BCGs
1	24658	present in 13 BCGs
1	26348	present in 13 BCGs
1	30877	present in 13 BCGs
1	34154	present in 13 BCGs
1	38259	present in 13 BCGs
1	39216	present in 13 BCGs
1	40649	present in 13 BCGs
1	44613	present in 13 BCGs
1	47260	present in 13 BCGs
1	47769	present in 13 BCGs
1	52501	present in 13 BCGs
1	52698	present in 13 BCGs
1	54157	present in 13 BCGs
1	57244	present in 13 BCGs
1	57981	present in 13 BCGs
1	58963	present in 13 BCGs
1	63420	present in 13 BCGs
1	64980	present in 13 BCGs
1	67616	present in 13 BCGs
1	68636	present in 13 BCGs

1	70277	present in 13 BCGs
1	72142	present in 13 BCGs
1	73033	present in 13 BCGs
1	73817	present in 13 BCGs
1	75560	present in 13 BCGs
1	120639	present in 13 BCGs
1	120810	present in 13 BCGs
1	9278	present in 13 BCGs
1	103346	present in 13 BCGs
1	103378	present in 13 BCGs
1	103554	present in 13 BCGs
1	103605	present in 13 BCGs
1	103744	present in 13 BCGs
1	132976	present in 13 BCGs
1	132994	present in 13 BCGs
1	133264	present in 13 BCGs
1	175506	present in 13 BCGs
1	103143	present in 13 BCGs
1	103264	present in 13 BCGs
1	103431	present in 13 BCGs
1	122097	present in 13 BCGs
1	122140	present in 13 BCGs
1	122143	present in 13 BCGs
1	122162	present in 13 BCGs
1	103141	present in 13 BCGs
1	103165	present in 13 BCGs

1	103344	present in 13 BCGs
1	103426	present in 13 BCGs
1	103589	present in 13 BCGs
1	103301	present in 13 BCGs
1	103209	present in 13 BCGs
1	103255	present in 13 BCGs
1	103361	present in 13 BCGs
1	103669	present in 13 BCGs
1	103716	present in 13 BCGs
1	103037	present in 13 BCGs
1	103078	present in 13 BCGs
1	103244	present in 13 BCGs
1	103464	present in 13 BCGs
1	121059	present in 13 BCGs
1	103198	present in 13 BCGs
1	103420	present in 13 BCGs
1	103055	present in 13 BCGs
1	103153	present in 13 BCGs
1	103531	present in 13 BCGs
1	103577	present in 13 BCGs
1	103536	present in 13 BCGs
1	103754	present in 13 BCGs
1	103010	present in 13 BCGs
1	103230	present in 13 BCGs
1	103493	present in 13 BCGs
1	103569	present in 13 BCGs

1	103738	present in 13 BCGs
1	103751	present in 13 BCGs
1	103004	present in 13 BCGs
1	103018	present in 13 BCGs
1	103021	present in 13 BCGs
1	103029	present in 13 BCGs
1	103090	present in 13 BCGs
1	103105	present in 13 BCGs
1	103137	present in 13 BCGs
1	103260	present in 13 BCGs
1	103286	present in 13 BCGs
1	103304	present in 13 BCGs
1	103349	present in 13 BCGs
1	103386	present in 13 BCGs
1	103415	present in 13 BCGs
1	103516	present in 13 BCGs
1	103547	present in 13 BCGs
1	103747	present in 13 BCGs
1	120410	present in 13 BCGs
1	120537	present in 13 BCGs
1	103452	present in 13 BCGs
1	120569	present in 13 BCGs
1	120912	present in 13 BCGs
1	120802	present in 13 BCGs
1	103274	present in 13 BCGs
1	103620	present in 13 BCGs

1	103694	present in 13 BCGs
1	103425	present in 13 BCGs
1	103434	present in 13 BCGs
1	103693	present in 13 BCGs
1	103128	present in 13 BCGs
1	103170	present in 13 BCGs
1	103121	present in 13 BCGs
1	103291	present in 13 BCGs
1	103347	present in 13 BCGs
1	103636	present in 13 BCGs
1	103641	present in 13 BCGs
1	103651	present in 13 BCGs
1	72741	present in 13 BCGs
1	120392	present in 13 BCGs
1	120452	present in 13 BCGs
1	121017	present in 13 BCGs
1	223	present in 13 BCGs
1	503	present in 13 BCGs
1	1545	present in 13 BCGs
1	2695	present in 13 BCGs
1	3400	present in 13 BCGs
1	6323	present in 13 BCGs
1	8688	present in 13 BCGs
1	18700	present in 13 BCGs
1	21096	present in 13 BCGs
1	21797	present in 13 BCGs

1	34776	present in 13 BCGs
1	40165	present in 13 BCGs
1	42790	present in 13 BCGs
1	43332	present in 13 BCGs
1	43514	present in 13 BCGs
1	45250	present in 13 BCGs
1	48646	present in 13 BCGs
1	49421	present in 13 BCGs
1	49872	present in 13 BCGs
1	50442	present in 13 BCGs
1	52026	present in 13 BCGs
1	56895	present in 13 BCGs
1	64079	present in 13 BCGs
1	67697	present in 13 BCGs
1	72314	present in 13 BCGs
1	72515	present in 13 BCGs
1	76584	present in 13 BCGs
1	103272	present in 13 BCGs
1	103457	present in 13 BCGs
1	103530	present in 13 BCGs
1	103532	present in 13 BCGs
1	103578	present in 13 BCGs
1	103668	present in 13 BCGs
1	103729	present in 13 BCGs
1	103732	present in 13 BCGs
1	103067	present in 13 BCGs

1	103283	present in 13 BCGs
1	103355	present in 13 BCGs
1	103479	present in 13 BCGs
1	103501	present in 13 BCGs
1	103537	present in 13 BCGs
1	20662	present in 13 BCGs
1	53355	present in 13 BCGs
1	56043	present in 13 BCGs
1	67156	present in 13 BCGs
1	103130	present in 13 BCGs
1	103180	present in 13 BCGs
1	103718	present in 13 BCGs
1	103731	present in 13 BCGs
1	103296	present in 13 BCGs
1	103338	present in 13 BCGs
1	103472	present in 13 BCGs
1	103548	present in 13 BCGs
1	103687	present in 13 BCGs
1	9474	present in 13 BCGs
1	9475	present in 13 BCGs
1	10022	present in 13 BCGs
1	20281	present in 13 BCGs
1	21497	present in 13 BCGs
1	39354	present in 13 BCGs
1	44231	present in 13 BCGs
1	53833	present in 13 BCGs

1	55157	present in 13 BCGs
1	55158	present in 13 BCGs
1	103393	present in 13 BCGs
1	103116	present in 13 BCGs
1	103197	present in 13 BCGs
1	103247	present in 13 BCGs
1	103162	present in 13 BCGs
1	103002	present in 13 BCGs
1	103118	present in 13 BCGs
1	103262	present in 13 BCGs
1	103267	present in 13 BCGs
1	103082	present in 13 BCGs
1	103746	present in 13 BCGs
1	103185	present in 13 BCGs
1	103246	present in 13 BCGs
1	103384	present in 13 BCGs
1	103476	present in 13 BCGs
1	103480	present in 13 BCGs
1	103490	present in 13 BCGs
1	103612	present in 13 BCGs
1	103629	present in 13 BCGs
1	103700	present in 13 BCGs
1	103125	present in 13 BCGs
1	103756	present in 13 BCGs
1	103017	present in 13 BCGs
1	103031	present in 13 BCGs

1	103179	present in 13 BCGs
1	103566	present in 13 BCGs
1	103677	present in 13 BCGs
1	103703	present in 13 BCGs
1	103112	present in 13 BCGs
1	103549	present in 13 BCGs
1	103717	present in 13 BCGs
1	103755	present in 13 BCGs
1	120587	present in 13 BCGs
1	120630	present in 13 BCGs
1	120878	present in 13 BCGs
1	120961	present in 13 BCGs
1	121057	present in 13 BCGs
1	103359	present in 13 BCGs
1	103403	present in 13 BCGs
1	103708	present in 13 BCGs
1	103102	present in 13 BCGs
1	103297	present in 13 BCGs
1	103432	present in 13 BCGs
1	103022	present in 13 BCGs
1	103469	present in 13 BCGs
1	103645	present in 13 BCGs
1	19562	present in 13 BCGs
1	103482	present in 13 BCGs
1	103664	present in 13 BCGs
1	103534	present in 13 BCGs

1	4132	present in 13 BCGs
1	8900	present in 13 BCGs
1	14574	present in 13 BCGs
1	35316	present in 13 BCGs
1	41071	present in 13 BCGs
1	47209	present in 13 BCGs
1	72294	present in 13 BCGs
1	103013	present in 13 BCGs
1	103695	present in 13 BCGs
1	120887	present in 13 BCGs
1	433	present in 13 BCGs
1	4334	present in 13 BCGs
1	4990	present in 13 BCGs
1	8464	present in 13 BCGs
1	25315	present in 13 BCGs
1	42633	present in 13 BCGs
1	58022	present in 13 BCGs
1	67607	present in 13 BCGs
1	71346	present in 13 BCGs
1	105967	present in 13 BCGs
1	105976	present in 13 BCGs
1	38439	present in 13 BCGs
1	46806	present in 13 BCGs
1	1522	present in 13 BCGs
1	3402	present in 13 BCGs
1	17838	present in 13 BCGs

1	39011	present in 13 BCGs
1	49333	present in 13 BCGs
1	51790	present in 13 BCGs
1	70010	present in 13 BCGs
1	98793	present in 13 BCGs
1	98906	present in 13 BCGs
1	103697	present in 13 BCGs
1	103176	present in 13 BCGs
1	103331	present in 13 BCGs
1	103510	present in 13 BCGs
1	103245	present in 13 BCGs
1	103340	present in 13 BCGs
1	103649	present in 13 BCGs
1	103208	present in 13 BCGs
1	103258	present in 13 BCGs
1	103511	present in 13 BCGs
1	120408	present in 13 BCGs
1	120428	present in 13 BCGs
1	98728	present in 13 BCGs
1	98784	present in 13 BCGs
1	98852	present in 13 BCGs
1	103284	present in 13 BCGs
1	103385	present in 13 BCGs
1	63850	present in 13 BCGs
1	25138	present in 13 BCGs
1	61020	present in 13 BCGs

1	20198	present in 13 BCGs
1	61340	present in 13 BCGs
1	55967	present in 13 BCGs
1	71325	present in 13 BCGs
1	7311	present in 13 BCGs
1	103339	present in 13 BCGs
2	103579	changed in 13 BCGs
2	103462	changed in 13 BCGs
2	103061	changed in 13 BCGs
2	103227	changed in 13 BCGs
2	9924	A point mutation in 13 BCGs
2	120511	A point mutation in 13 BCGs
2	103365	2 SNPs in 13 BCGs
2	103503	2 SNPs in 13 BCGs
2	103007	3 SNPs in 13 BCGs
2	18898	A point mutation in 13 BCGs
2	10841	A point mutation in 13 BCGs
2	103266	A point mutation in 13 BCGs
2	120892	A point mutation in 13 BCGs
2	103352	A point mutation in 13 BCGs
2	103038	9 SNPs in 13 BCGs
3	120399	lost in 13 BCGs
3	21765	lost in 13 BCGs
3	27635	lost in 13 BCGs
3	41948	lost in 13 BCGs
3	66693	lost in 13 BCGs

3	4556	lost in 13 BCGs
3	187	lost in 13 BCGs
3	655	lost in 13 BCGs
3	3934	lost in 13 BCGs
3	21422	lost in 13 BCGs
3	28505	lost in 13 BCGs
3	32971	lost in 13 BCGs
3	33831	lost in 13 BCGs
3	34927	lost in 13 BCGs
3	37040	lost in 13 BCGs
3	41028	lost in 13 BCGs
3	50549	lost in 13 BCGs
3	103519	lost in 13 BCGs
3	120421	lost in 13 BCGs
3	60	lost in 13 BCGs
3	35139	lost in 13 BCGs
3	46500	lost in 13 BCGs
3	52048	lost in 13 BCGs
3	61578	lost in 13 BCGs
3	120550	lost in 13 BCGs
3	120870	lost in 13 BCGs
4	120441	lost in 8 BCGs
4	120788	lost in 8 BCGs
4	22997	lost in 8 BCGs
4	24522	lost in 8 BCGs
4	44381	lost in 8 BCGs

4	103145	lost in 8 BCGs
4	103525	lost in 8 BCGs
4	103635	lost in 8 BCGs
4	103661	lost in 8 BCGs
4	103327	lost in 8 BCGs
4	103348	lost in 8 BCGs
4	103686	lost in 8 BCGs
4	120777	lost in 8 BCGs
5	41952	lost in BCG-Swedens
5	53107	lost in BCG-Phipps
5	53303	5 base deletion in BCG-Phipps
5	73020	2 base deletion in BCG-Phipps
5	48905	11 base deletion in BCG-Phipps
5	6825	8 base deletion in BCG-Phipps
5	49246	14 base deletion in BCG-Phipps
5	55523	lost in BCG-Phipps
5	55706	17 base deletion in BCG-Phipps
5	94762	A point mutation in BCG-Moreau, BCG-Russia and BCG-Tice, 3 SNPs and 3 base deletion in BCG-Prague
5	120904	lost in BCG-Phipps, 21 base deletion in BCG-Prague
5	22387	A point mutation in BCG-Phipps, BCG-Prague, BCG-Russia, BCG-Tice
5	103371	A point mutation in BCG-Glaxo
5	103633	Single-base insert in BCG-Phipps
5	7472	Single-base insert in BCG-Phipps
5	103659	Single-base insert in BCG-Phipps
5	120481	Single base deletion in BCG-Moreau

5	103373	Single base deletion in BCG-Moreau
5	103737	Single base deletion in BCG-Moreau
5	103000	17 base deletion in BCG-Prague
5	103199	8 base deletion in BCG-Prague
5	103720	Single base deletion in BCG-Moreau
5	103195	Single base deletion in BCG-Moreau
